# Area-based social inequalities in adult mortality: construction of French deprivation-specific life tables for the period 2016–2018

**DOI:** 10.3389/fpubh.2023.1310315

**Published:** 2023-12-19

**Authors:** Ophélie Merville, Quentin Rollet, Olivier Dejardin, Ludivine Launay, Élodie Guillaume, Guy Launoy

**Affiliations:** ^1^U1086 “ANTICIPE” INSERM Labelled ≪ Ligue Contre le Cancer ≫, Centre François Baclesse, University of Caen Normandie, Caen, France; ^2^Inequalities in Cancer Outcomes Network (ICON), Department of Non-Communicable Disease Epidemiology, Faculty of Epidemiology and Population Health, London School of Hygiene and Tropical Medicine, London, United Kingdom

**Keywords:** life-tables, deprivation, health inequalities, ecological index, all-cause mortality

## Abstract

**Background:**

In order to tackle social inequalities in mortality, it is crucial to quantify them. We produced French deprivation-specific life tables for the period 2016–2018 to measure the social gradient in adult all-cause mortality.

**Methods:**

Data from the Permanent Demographic Sample (EDP) were used to provide population and death counts by age, sex and deprivation quintile. The European Deprivation Index (EDI), applied at a sub-municipal geographical level, was used as an ecological measure of deprivation. Smoothed mortality rates were calculated using a one-dimensional Poisson counts smoothing method with P-Splines. We calculated life expectancies by age, sex and deprivation quintile as well as interquartile mortality rate ratios (MRR).

**Results:**

At the age of 30, the difference in life expectancy between the most and least deprived groups amounted to 3.9 years in males and 2.2 years in females. In terms of relative mortality inequalities, the largest gaps between extreme deprivation groups were around age 55 for males (MRR = 2.22 [2.0; 2.46] at age 55), around age 50 in females (MRR = 1.77 [1.48; 2.1] at age 47), and there was a decrease or disappearance of the gaps in the very older adults.

**Conclusions:**

There is a strong social gradient in all-cause mortality in France for males and females. The methodology for building these deprivation-specific life tables is reproducible and could be used to monitor its development. The tables produced should contribute to improving studies on net survival inequalities for specific diseases by taking into account the pre-existing social gradient in all-cause mortality.

## 1 Introduction

Over the last few decades, life expectancy at birth has increased overall for all strata of the population, but a social gradient in mortality persists and has even increased in some regions of the world (1, 2). Many studies have highlighted the association of the socio-economic status of individuals or their living environment with all-cause and cause-specific mortality (1, 3–14). In Europe, these inequalities are also observed, even in developed countries with effective, protective health systems (15–17).

National life tables updated over time and stratified by age and sex are very useful health policy tools and provide global health indicators such as life expectancy. In order to tackle these social inequalities in mortality, it is crucial to quantify them. The inclusion of deprivation level as a health determinant, along with age or sex, is relevant for studying the social gradient in all-cause mortality, and such deprivation-specific life tables are already available for several European countries (3, 8, 18). The use of a cross-national measure of deprivation, such as the European Deprivation Index (EDI), which allows comparisons between countries, makes life tables even more relevant.

In several countries such as France, socio-economic data are either unavailable or are scanty at the individual level in databases (19), so ecological indices of deprivation are widely used to study social health inequalities. These indices can be used as a proxy for individual deprivation (20) or to measure the level of deprivation in a geographical area (21). When using these indices to approximate the level of deprivation of individuals, it is recommended to use the smallest measurement grid available to limit ecological bias (22). When used as a contextual measure, it may also be appropriate to apply these indices at a fine geographical level if the focus is on the neighborhood.

When investigating social inequalities in survival for a specific disease such as cancer, it is also important to account for the pre-existing social gradient in all-cause mortality. If this ‘background mortality' is not taken into account in the analyses, there is a risk of overestimating the excess mortality related to social inequalities for this specific disease (6, 23). Given the lack of appropriate estimation of life expectancy according to social status or deprivation, authors have to simulate it using fragile hypotheses and experimental solutions (24, 25) or extract the social gradient from life tables of other countries, which could introduce a large bias in the analyses (6, 23, 26). The availability of national life tables according to social status or deprivation makes it possible to measure accurately the social gradient in excess mortality related to a specific disease (1, 7, 8, 14).

In this study, we aimed to produce French deprivation-specific life tables for the period 2016–2018 by focusing on adults living in Metropolitan France and by using an ecological index of deprivation applied at a sub-municipal level. Our objectives were (a) to provide an overview of inequalities in all-cause mortality for a recent period by proposing a reproducible method, and (b) to create French life tables crucial for future studies on excess mortality related to social inequalities regarding specific diseases.

## 2 Methods

### 2.1 Mortality and population data

Data from the Permanent Demographic Sample (EDP) were used to provide population and death counts by age and level of deprivation. The EDP corresponds to a large panel of the French population sampled by day of birth (EDP days). This system collects information from different sources of official statistics over time. The EDP was set up in 1968 and, since 2008, has covered about 4% of the French population with 16 EDP days spread over the year. EDP individuals are followed-up across census and civil statistics for every demographic event recorded such as birth, marriage or death. In France, the population census is carried out on a five-year rotating basis with differences in the composition of the census units depending on the size of the municipality. At the end of a five-year cycle, the five annual census surveys (ACS) provide population data for all small municipalities (fewer than 10,000 inhabitants) and for 40% of the large ones (more than 10,000 inhabitants).

**Figure 1:**
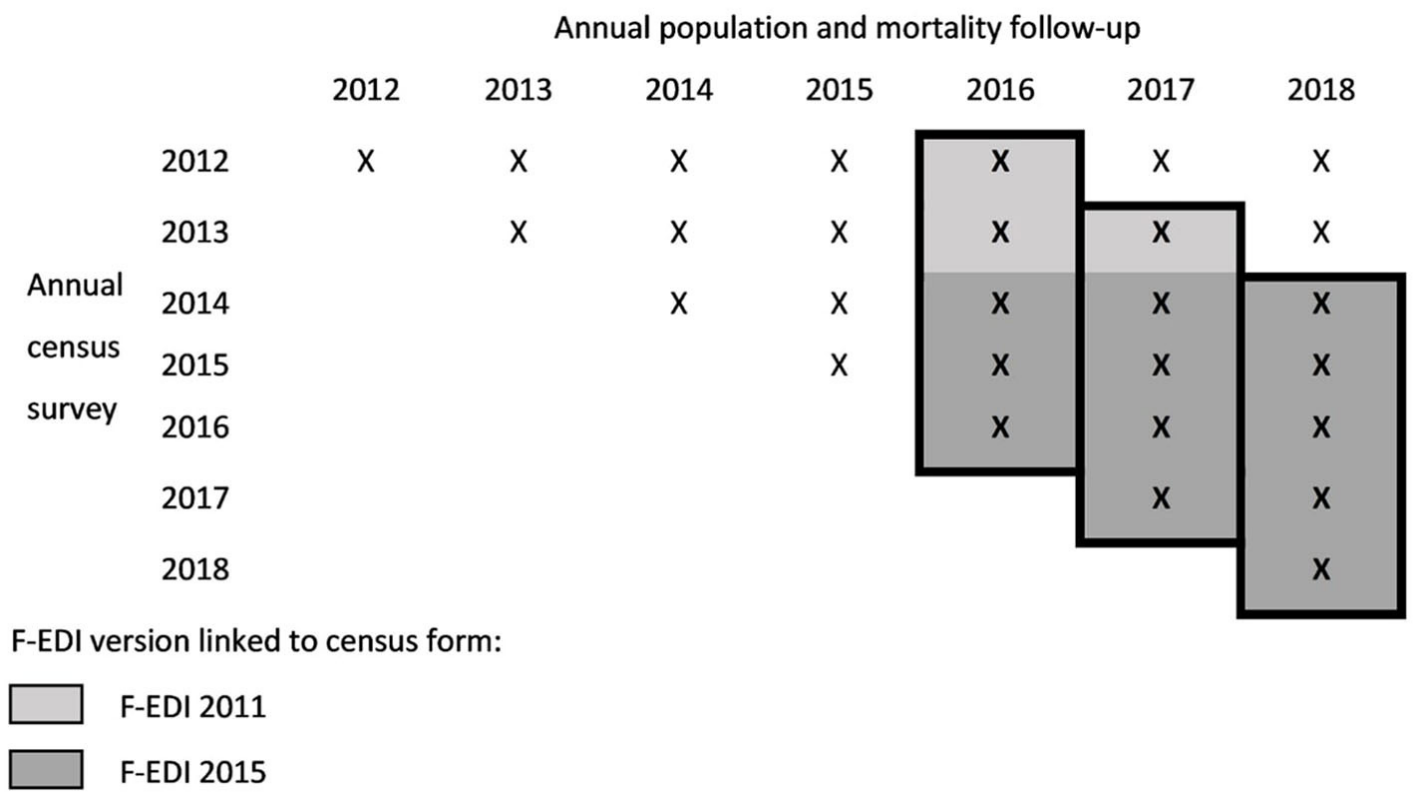
3-year mortality follow-up (from 2016 to 2018) using the annual census survey and mortality records.

To construct deprivation-specific life tables, only individuals in the EDP with a census form were selected. Given the features of the French census, the data were organized to take into account the annual population and mortality records of the EDP individuals followed in the previous five ACS samples. For example, mortality and population data for the year 2016 were collected among the surviving EDP individuals tracked in the ACSs 2012 to 2016 (Figure 1). If an EDP individual was surveyed more than once during this five-year period, we selected the most recent census form.

As with the national census data, there was an over-estimation of individuals living in small municipalities in the study population and weights—survey weights centered on 1 for each ACS—were used to correct for this bias. To increase the power of the results, 3 years of population and mortality follow-up data were considered, from 2016 to 2018, to produce life tables. EDP individuals under the age of 18 were excluded because the number of deaths at younger ages was too low, resulting in unreliable estimates.

Thus, information on sex, age at follow-up and death—single year of age—were extracted from the civil status records for each year of follow-up and census forms were used to provide a level of deprivation. We then summed the number of deaths and population for the 3 years of follow-up from 2016 to 2018 to calculate estimates of mortality rates by age, sex and level of deprivation.

### 2.2 Deprivation measure

The French version of the European Deprivation Index (F-EDI) was used as deprivation measure. The EDI was built using a methodology proposed by Pornet et al. (27) and then applied to several European countries (28). The F-EDI is derived from a weighted combination of aggregate national census variables that are most highly correlated with deprivation markers identified from the European Union Statistics on Income and Living Conditions (EU-SILC) survey. Depending on the census year, we used the 2011 version of the F-EDI or the 2015 updated version (Figure 1). The 2011 version includes the percentage of the following: non-owners, overcrowded housing, unskilled workers, residents with low education, residents of foreign nationality, unemployed, single-parent households, households without access to a car, households with six or more persons, and households without a bath or shower. For the 2015 version, the last two variables are replaced by the percentage of households with two or more persons and of unmarried residents. An F-EDI score was calculated for each residential IRIS (Ilôt Regroupés pour l'Information Statistique) in Metropolitan France, i.e., the smallest geographical unit for which French census data from 2011 or 2015 were available.

For each individual in the sample, data from their most recent individual census form was used to assign them an F-EDI score according to their IRIS of residence. The population was divided into five categories of deprivation using the five national quintiles of the F-EDI score, from least deprived (q1) to most deprived (q5). To create these national quintiles, we considered the IRIS population in 2011 or 2015, depending on the version of the index, so that each quintile corresponded to around 20% of the Metropolitan French population.

### 2.3 Statistical analysis

A method for smoothing one-dimensional Poisson counts with P-Splines was used to calculate smoothed mortality rates (29). Male and female death counts were modeled separately assuming a smooth mortality trajectory over age (x from 18 to 100).

Estimates of the number of deaths by age and sex obtained from the EDP sample to predict mortality rates were recalibrated on the basis of national life tables produced by INSEE (Institut national de la statistique et des études économiques) for the same period (available at https://www.insee.fr/fr/statistiques/6327207?sommaire=6327254&q=tables+de+mortalit%C3%A9). The mortality rate estimates produced without recalibration were compared with data produced by INSEE and presented in Supplementary material 1.

We then stratified analyses by level of deprivation assuming independent estimates for each deprivation quintile and applying the calibration coefficient obtained from the full sample separately for males and females.

Deaths were considered as realizations of a Poisson distribution where the expected values (*d*_*x*_) are the product of the exposures (*e*_*x*_) and the force of mortality (*u*_*x*_):


$$\begin{array}{l} d_{x}\sim \;Poi\left(e_{x}\;*\;\mu_{x}\right) \end{array}$$

Poisson data were modeled using a generalized linear model with equally spaced cubic B-Splines as regression basis, a log link function of Poisson death counts and person-years at risk as the offset i.e.:


$$\begin{array}{l} \log\left(E\left(d_{x}\right)\right)=\log\left(e_{x}\right)+\log\left(\mu_{x}\right)=\log\left(e_{x}\right)+B_{a_{x}} \end{array}$$

where *a*_*x*_ are the coefficients associated with each B-Spline.

The P-Splines approach consisted of adding a roughness penalty on the regression coefficients to the combination of B-Spline basis. The smoothing parameter controlled the trade-off between smoothness and model fidelity and was selected to minimize the Bayesian Information Criterion (BIC) of the model.

As detailed by Eilers and Marx (30), the number of knots is not as important as the choice of the smoothing parameter but should be large enough to ensure that all possible features in the data are eventually captured. To set the number of knots, different options were tested, with several knots per decade, without significantly affecting the results. The intermediate option was chosen and the number of knots was set at 21 (dividing the age range by 4 to set this value).

Mortality rates and their 95% confidence intervals (CI) were predicted from the fitted models by age, sex and deprivation quintile. Life expectancies were calculated from the mortality quotients obtained via the mortality rates.

Interquartile mortality rate ratios (MRR) were calculated for males and females by age, using the least deprived group (q1) as reference. To produce the 95% CI of the MRR, we used the delta method, i.e., a method for computing the variability of a function of estimates. In our case, the function corresponded to the difference between quintile-specific linear predictors.

All calculations were performed using R software version 4.0.2 (R Core Team, Vienna, Austria), the MortalitySmooth R package, specifically tailored to mortality data (29), was used for smoothing mortality rates, and the LifeTableFUN.R script (available at https://sites.google.com/site/carlogiovannicamarda/r-stuff?authuser=0) was used to build the life tables.

## 3 Results

We used data of 1,668,533 EDP individuals (806,281 males and 862,252 females) and 50,793 deaths were identified in this population (25,337 in males and 25,456 in females).

**Figure 2:**
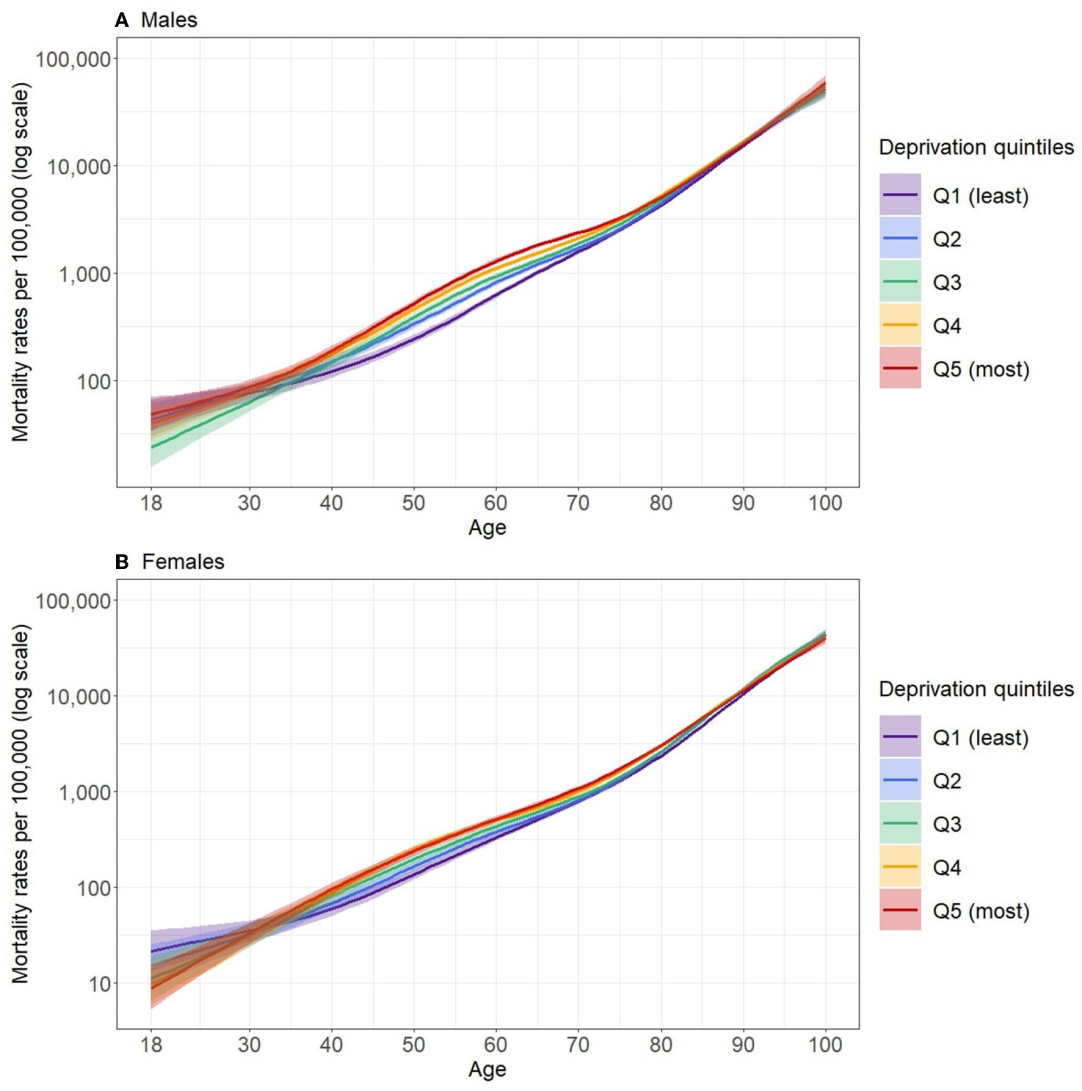
Predicted mortality rates (log scale) according to quintiles of deprivation for the period 2016–2018 in males **(A)** and females **(B)** in Metropolitan France.

The predicted mortality rates (and their 95% CI) by single year of age, sex, F-EDI quintile and for all levels of deprivation are presented in Supplementary material 2. For both sexes, mortality rates increased from age 18–100, regardless of the deprivation quintile (Figure 2). A social gradient in mortality by deprivation quintile was observable particularly in the middle of the age range, with higher mortality rates for the most deprived quintiles.

**Figure 3:**
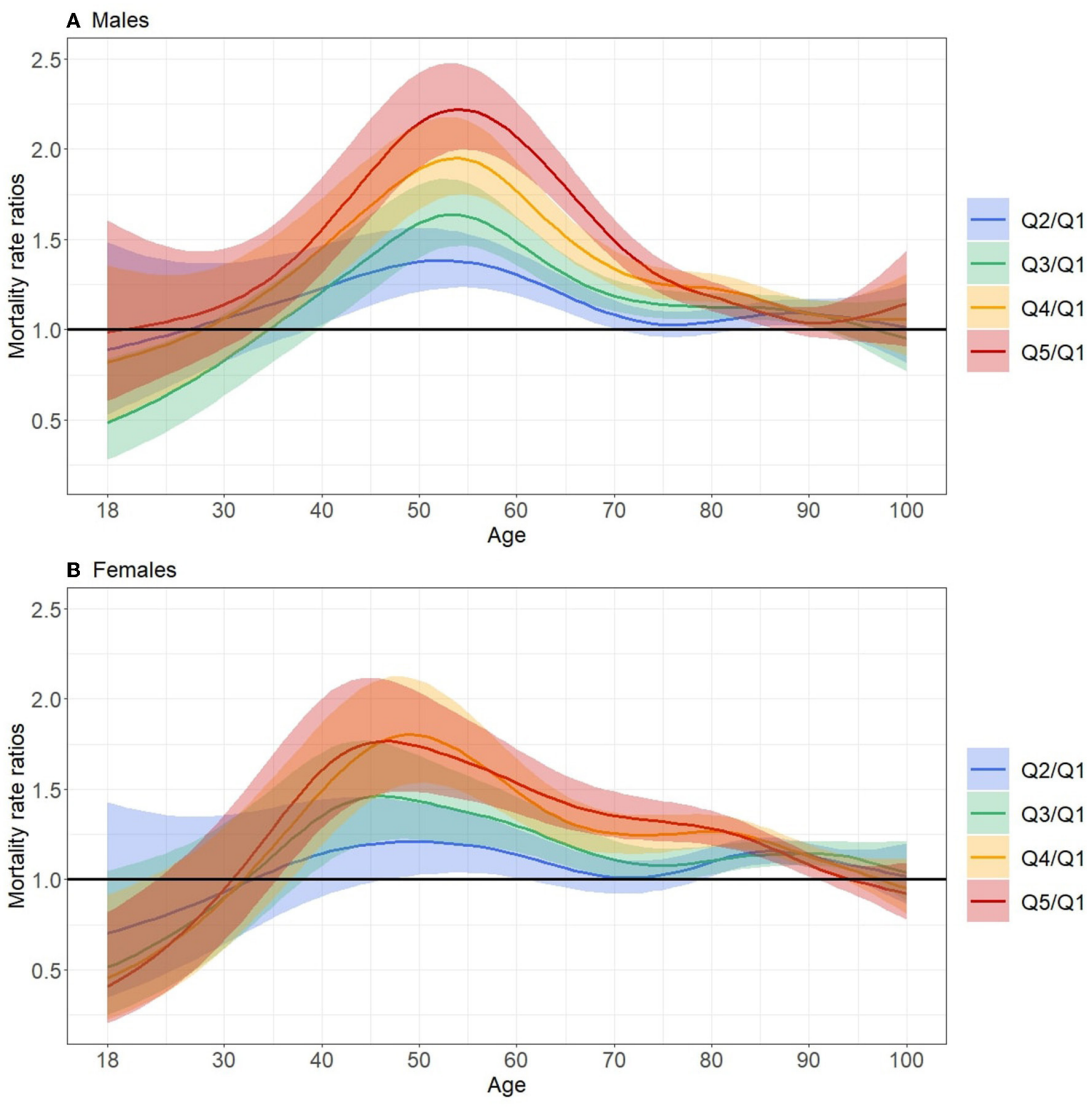
Mortality rate ratios as function of age between quintiles Q2, Q3, Q4 and Q5 and least deprived quintile (Q1) for the period 2016–2018 in males **(A)** and females **(B)** in Metropolitan France.

The largest relative inequalities in mortality between the least and most deprived quintiles occurred around age 55 for males (MRR = 2.22 [2.0; 2.46] at age 55) and around age 50 in females (MRR = 1.77 [1.48; 2.1] at age 47), as shown in Figure 3 (data available in Supplementary material 3). After this peak, MRR decreased and no significant differences between deprivation quintiles were observed from about 85 years of age for both males and females. For younger people, the relative inequalities appeared to be largely reduced and the width of the 95% CI did not allow significant differences between the deprivation quintiles to be distinguished.

Overall, there were greater inequalities for males. The gradient was less pronounced in females, where there was no significant gap between the two most deprived quintiles (q4–q5). In addition, we observed a lag in the onset of mortality inequalities by sex, with relative inequalities starting earlier in females.

**Figure 4:**
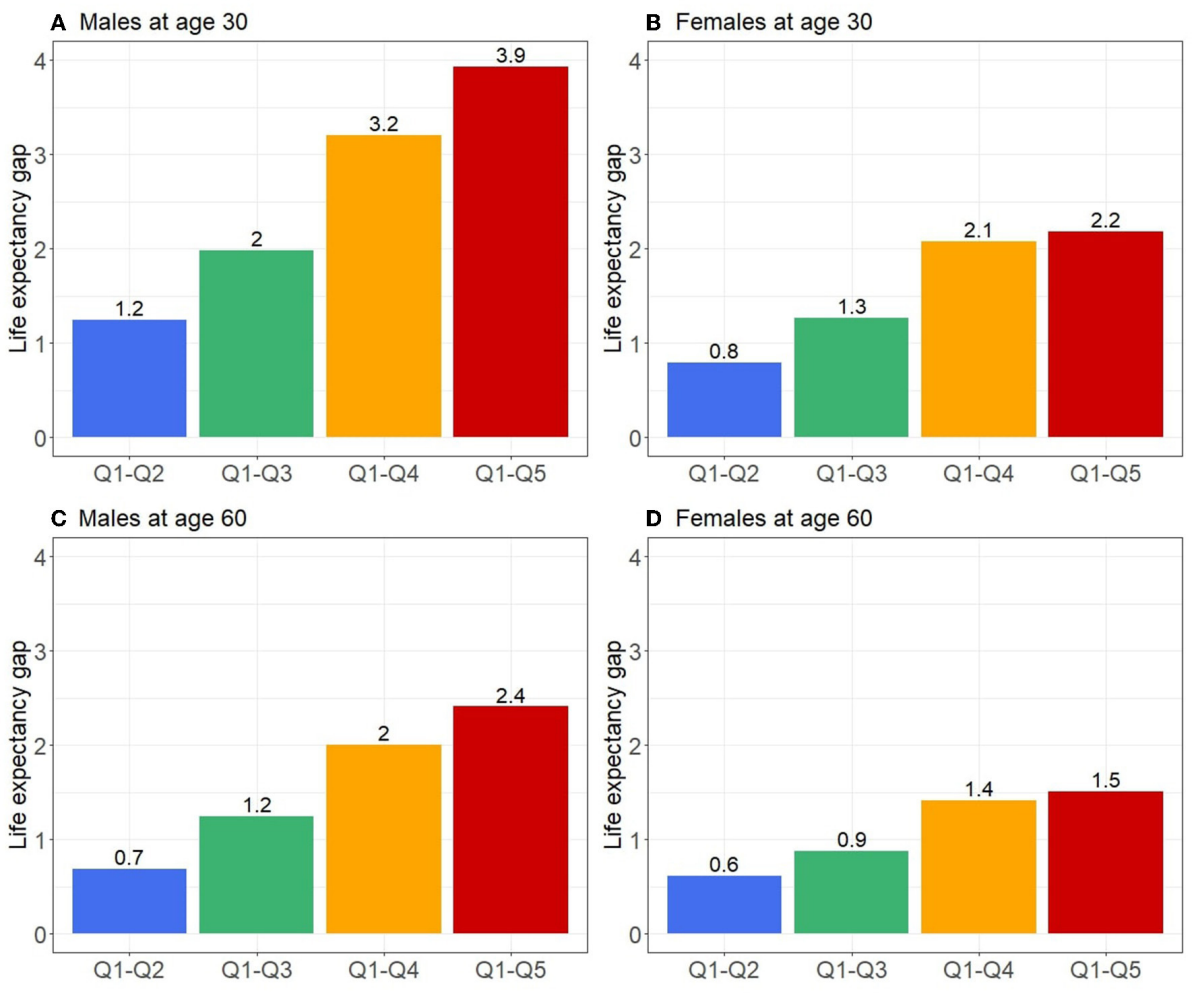
Life expectancy gap at age 30 and 60 for males **(A, C)** and females **(B, D)** between the least deprived quintile (Q1) and the other quintiles (Q2–Q5) for the period 2016–2018 in Metropolitan France.

The deprivation-specific life tables for the period 2016–2018, available as supplementary data (Supplementary material 2), present life expectancies calculated from mortality rates by age, sex and deprivation quintiles. For males, there was a gap of 3.9 years at age 30 between the extreme quintiles, which then decreased with age, with a gap of 2.4 years at age 60 (Figure 4). The same dynamic was observed, but to a lesser extent for females with a gap of 2.2 years at age 30 and 1.55 at age 60.

## 4 Discussion

For Metropolitan France over the period 2016–2018, we observed a social gradient in mortality in both sexes with greater inequalities in males. At the age of 30, the difference in life expectancy between the most and least deprived groups amounted to 3.9 years in males and 2.2 years in females, before decreasing over the years. In terms of relative mortality inequalities, the largest gaps between deprivation groups were around age 55 for males and 5 years earlier in females, and there was a decrease or disappearance of the gaps in the older adults.

As expected, we observed higher mortality rates and lower life expectancy in males than in females at all ages (1, 3, 8). There were also stronger absolute and relative inequalities in all-cause mortality in males. Most studies found the same trend persisting over time (1, 3, 7, 8, 10, 11, 13), with some authors explaining this larger gap in males by differences in the pattern of causes of death (4, 11, 12). Studies have particularly highlighted the role of external causes of death such as accidents, violence, drug and alcohol abuse, which are more common among males (4, 11, 12).

Regarding relative inequalities, the study by Antunes et al. using Portuguese national data showed a maximum social gradient at birth that decreased with age (3). In other studies, there was an initial peak in early childhood, a second peak in middle age, and then a disappearance of the gradient in the older adults (1, 10, 13). In the present study, data for the under-18s could not be exploited, but a similar pattern was observed with maximum MRR values between the extreme deprivation groups around age 55 in males and 50 in females. However, we did not find anything in the literature to explain the 5-year gap between males and females reported in our study, so this difference requires further investigation.

Caution is required when comparing the magnitude of inequalities observed between deprivation groups with the results of previous studies because of their heterogeneity in statistical methodology, the deprivation indicator used, the time scale, and the size of the geographical units used with an ecological index. In addition, there are differences between health systems and populations in different countries have different specificities. For example, using Portuguese national data for the period 2010–2012, Antunes et al. found a gap in life expectancy at age 30 of 1.7 years in males and 0.8 years in females between the most and least deprived groups (3). Mayhew et al. found a much larger gap with 8.6 years in males and 6.6 years in females using English national data for 2016 (1). The two studies used different indices of deprivation applied at sub-municipal level and the former used quintiles while the latter used deciles, which may partly explain the larger gaps observed. In addition, studies have already shown that there is a lower social gradient in mortality in southern Europe (31). In our study, we found intermediate results with a gap in life expectancy at 30 years of 3.9 years in males and 2.2 years in females between the most and least deprived quintiles (Supplementary material 2).

Several methods have been developed to estimate complete life tables, mainly using aggregate data such as age groups. Rachet et al. designed a modeling approach to estimate smoothed mortality rates using flexible Poisson multivariable models that has already been used to build deprivation-specific life tables (3, 8, 32). For this study, we applied another methodological approach to smoothing Poisson counts with P-Splines that was developed by Camarda (29) and has also been used by others (5).

In this study, individuals under the age of 18 were excluded due to the small number of deaths at young ages, which made our estimates unreliable. However, even when focusing on individuals aged 18 and over, the number of deaths remained low before the age of 30. The MRR values should therefore be interpreted with caution to reveal a possible social gradient in mortality due to the width of the confidence intervals for younger ages. This limitation was expected and several previous studies on adult mortality focused on individuals aged 30 years and older (1, 9).

Individuals living in the French overseas departments were excluded because of the controversy surrounding the use of the F-EDI outside Metropolitan France. The French sample of the EU-SILC survey used as a basis for constructing the index does not include individuals living in these departments, which nevertheless present significant specificities, with a much higher level of poverty, particularly in Mayotte and French Guyana, than in Metropolitan France (33). The census forms also include questions on living conditions specific to these departments which are not taken into account in the construction of the F-EDI. The lack of a validated deprivation index for these areas is a major obstacle to studying social health inequalities across the whole of France. The almost systematic exclusion of the overseas departments from the analyses does not allow for the identification of any specific needs and their consideration in public health actions and further studies are crucial to fill this gap.

For this study, it was assumed that individuals had not moved and that the place of residence reported on the census form was the same in the year of population and mortality follow-up, despite a time lag of up to 4 years. Furthermore, by including individuals whose census date was in the year of death, potential moves related to a deterioration in health status leading to death in the short term were not taken into account. The decision to use a 5-year period for the selection of census forms to keep abreast with the rotation cycle of the French census seemed to be a good compromise between limiting the anteriority of the information and maximizing the number of deaths. In addition, by using this methodology, we did not take residential mobility into account or potential changes in social environment over the life course that could have an impact on the health of individuals. In a population with diverse ethno-racial origins, as in metropolitan France, this limitation does not allow us to consider the potential impact of migration on mortality for example. However, a previous study examining the link between socioeconomic environment and cancer incidence showed that, although the impact of misclassification induced by the residential mobility was substantial, it remained non-differential and conservative (34).

We used two versions of the EDI-−2011 and 2015—for the analyses according to the census year. Both versions of the index follow exactly the same construction methodology and their differences are supposed to illustrate the evolution of society in defining deprivation. The distributions of these scores are not exactly identical but the use of quintiles avoided this bias.

Most of the available national deprivation-specific life tables have been constructed from population-wide data. In France, death certificates contain little or no information on the social position of the deceased and on the last place of residence. For geographical data, only the municipality of the last known residence is indicated. To build specific life tables using an index of deprivation applied at sub-municipal level, it is necessary to use a sample of the French population for which individuals can be located at the appropriate geographical level and deaths counted. This can be done with the EDP by merging census forms with birth and death certificates. To generalize the results to the Metropolitan French population, the sample had to be representative of this population in terms of composition and location in Metropolitan France. The use of weights theoretically corrected the representativeness deficiencies related to the census methodology. Despite this, when comparing the mortality rates estimated from the EDP data, without stratification on the level of deprivation, to those from the full national data, we observed a slight underestimation of the values with our sample (Supplementary material 1). We therefore recalibrated our estimates on the national data produced by INSEE and assumed that the calibration coefficient obtained could be applied to each quintile of deprivation.

Despite some limitations, this study has several strengths. First, it provides a quantification of social inequalities in all-cause mortality for a recent period in Metropolitan France using an area-level measure of deprivation. Second, the methodology developed for this study is reproducible as long as the data are available. Thus, the evolution of this social gradient of all-cause mortality over time could be monitored, making it possible to assess the impact on these inequalities of various events such as a pandemic or national public health measures. Third, the deprivation index used, i.e., the EDI, provides a comparable measure of deprivation across European countries. This is a significant advantage when making international comparisons of social inequalities in mortality. Finally, to our knowledge, there are no specific French life tables using deprivation measured at sub-municipal level. This study fills this gap and should notably improve the assessment of the part of excess mortality due to social inequalities for specific diseases. Using the life tables available in the Supplementary file, it is possible to extract a social gradient by relating the mortality rates of each quintile to the overall sample rate for each age, and then applying this gradient to the unstratified national deprivation data. However, depending on the period studied, there may be a time lag to consider, and it may be necessary to extrapolate the gradient to younger ages, possibly before age 30.

## 5 Conclusion

Inequalities in all-cause mortality persist in France and further research is required to understand the underlying causes and to enable the development of effective public health policies. These future studies should use other variables to assess the level of disadvantage of individuals, notably at the individual level, and also vary the time periods for producing estimates by widening them to increase the robustness of the results or narrowing them to study annual variations more accurately. In this paper, we propose a methodology to produce French-specific deprivation life tables that provide information on all-cause mortality inequalities and a tool that should lead to a better understanding of the excess mortality due to deprivation for different diseases.

## Data availability statement

The original contributions presented in the study are included in the article/Supplementary material, further inquiries can be directed to the corresponding author.

## Author contributions

OM: Conceptualization, Formal analysis, Methodology, Software, Validation, Writing—original draft, Writing—review & editing. QR: Methodology, Software, Validation, Writing—review & editing. OD: Validation, Writing—review & editing. LL: Resources, Writing—review & editing. ÉG: Supervision, Validation, Writing—review & editing. GL: Conceptualization, Supervision, Validation, Writing—review & editing.
