# Supplementary material for: Area-based social inequalities in adult mortality: construction of French deprivation-specific life tables for the period 2016–2018
Source: Front Public Health. 2023 Dec 19;11:1310315. doi: 10.3389/fpubh.2023.1310315 (PMC10762790; doi:10.3389/fpubh.2023.1310315)
Supplement: Supplementary file 1 [file Table_1.DOCX]

Supplementary Material

# Supplementary Data

Additional_file2. Life tables by deprivation quintile (QEDI) for males and females in the EDP sample for the period 2016-2018 (mx – mortality rate at age x; qx – probability of dying between ages x and x+1; lx – number surviving to age x; ex – life expectancy at age x).

Additional_file3. Mortality rate ratios (d.mx) by deprivation quintile, using quintile 1 (Q1) as reference, for males and females in the EDP sample for the period 2016-2018..

# Supplementary Figures and Tables

Additional_file1. Predicted mortality rates from EDP data, without recalibration, and published INSEE mortality rates for the period 2016-2018 in males and females in Metropolitan France (log scale).

**
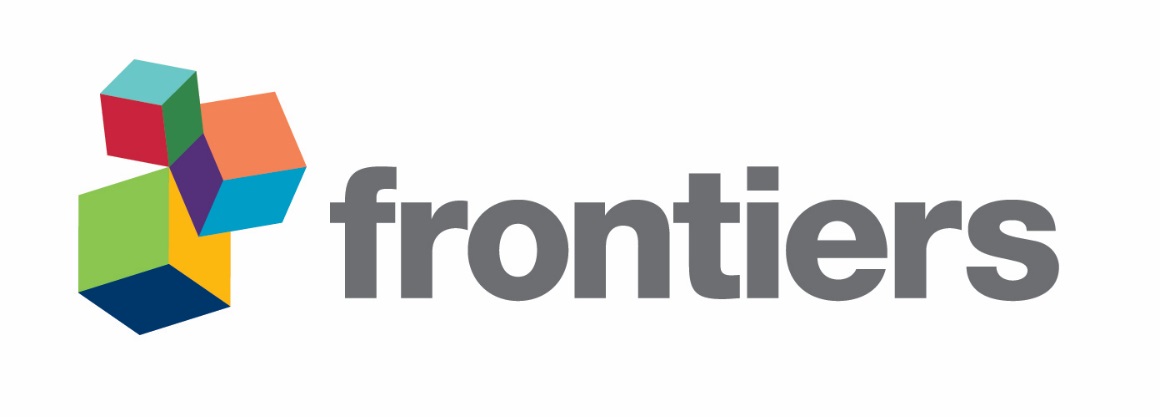
**
