# Supplementary figures and images for: Area-based social inequalities in adult mortality: construction of French deprivation-specific life tables for the period 2016–2018
Source: Front Public Health. 2023 Dec 19;11:1310315. doi: 10.3389/fpubh.2023.1310315 (PMC10762790; doi:10.3389/fpubh.2023.1310315)

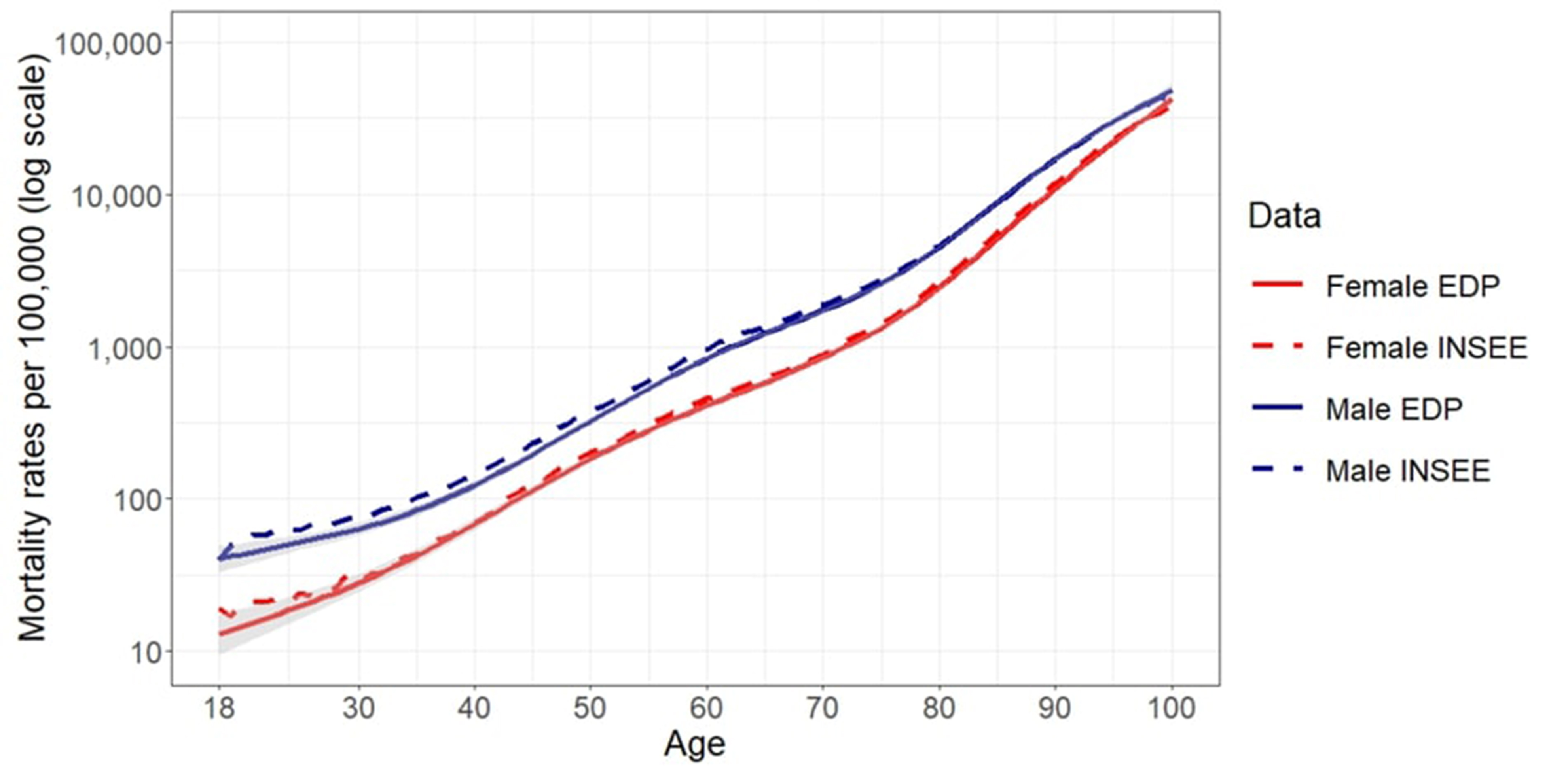

Supplement: Supplementary file 4 [file Image_1.JPEG]
